# The Role of Vitamins A and E Level in Chronic Suppurative Otitis Media with and without Cholesteatoma

**DOI:** 10.2147/JMDH.S414115

**Published:** 2023-11-10

**Authors:** Shinta Fitri Boesoirie, Wijana Hasansulama, Lina Lasminingrum, Arif Tria Novianto, Vesara Ardhe Gatera, Nur Akbar Aroeman, Thaufiq Siddiq Boesoirie

**Affiliations:** 1 Department of Otorhinolaryngology-Head and Neck Surgery Faculty of Medicine Universitas Padjadjaran/Dr. Hasan Sadikin General Hospital, Bandung, Indonesia; 2 Department of Pharmacy and Health Sciences, Universiti Kuala Lumpur – Royal College of Medicine Perak, Ipoh, Perak, Malaysia; 3 Center of Excellence in Higher Education for Pharmaceutical Care Innovation, Universitas Padjadjaran, Bandung, Indonesia

**Keywords:** antioxidants, cholesteatoma, CSOM, ROS, vitamin A, vitamin E

## Abstract

**Aim:**

High expression of lytic enzymes and cytokines is related to cell proliferation in Otitis Media Chronic Suppurative (CSOM) with cholesteatoma. In addition, the process of inflammation healing and maintenance of homeostatic conditions requires Reactive Oxygen Species (ROS), which can cause significant damage to cells. To address this issue, secondary antioxidants such as Vitamins A and E are used to inhibit and neutralize the occurrence of oxidation reactions. These vitamins complement each other, with vitamin A working effectively at low concentrations of oxygen, while vitamin E functions in the opposite manner.

**Purpose:**

This study aims to investigate the roles of vitamin A and E levels in CSOM patients with Cholesteatoma.

**Patients and Methods:**

The study was conducted between July and December 2020, and the method used was an analytical observational approach with a case–control design. The sample population comprised 60 CSOM patients divided into 2 groups. These included those with and without cholesteatoma.

**Results:**

The results showed that there was no significant difference between these two groups in terms of the impact of vitamin A and E levels (respectively, p = 0.626, p = 0.864).

**Conclusion:**

Considering these results, it was discovered that vitamins A and E levels do not influence the occurrence of CSOM with or without cholesteatoma.

## Introduction

Otitis media (OM) is defined by persistent infectious and inflammatory diseases of the middle ear and/or mastoid cavity.^1^,^2^ When the perforation persists and is accompanied by continuous or intermittent discharge for more than 2–6 weeks, this condition can advance into chronic suppurative otitis media (CSOM), with or without the presence of a cholesteatoma.^3–5^ CSOM with cholesteatoma specifically involving deposition around the inflamed tissues inside the ears is diagnosed as CSOM with cholesteatoma, and a cholesteatoma will develop to scrape the middle ear and the mastoid bone.^6–8^

CSOM with cholesteatoma can lead to bone damage in the tympanic cavity, mastoid, and nearby areas, increasing the risk of intratemporal and intracranial complications.^9^,^10^

An imbalance between increased reactive oxygen species (ROS) and antioxidant defence mechanisms triggers oxidative stress, causing cell and tissue damage. This disrupts wound healing process, promoting chronic inflammation that supports the formation of cholesteatoma in CSOM.^11^,^12^ Damage caused by free radicals in the body can be overcome by endogenous antioxidants. However, when the amount in the body exceeds the limits of endogenous antioxidant capabilities, exogenous antioxidants such as vitamin A and E are needed.^13^,^14^ This is because they play an essential role in binding free radicals and preventing chain oxidation reactions.^14^ Increased oxidative stress is presumably associated with decreased antioxidant levels, hence, treatment using vitamins has great potential for preventing and treating CSOM patients.^15^,^16^

Treatment developments for CSOM are evolving to enhance their effectiveness and safety, whether through medical therapy or surgical interventions. While there exists theoretical reasoning and some evidence suggesting that the levels of vitamins A and E may influence the incidence of cholesteatoma, studies on the role of these vitamins in CSOM patients with cholesteatoma remain limited. Therefore, this study aimed to examine the impact of vitamin A and E levels in CSOM with and without cholesteatoma. The results obtained can potentially serve as vital information for the development of subsequent therapeutic strategies aimed at reducing the occurrence of complications in CSOM patients.

## Materials and Methods

### Subjects

**Figure 1:**
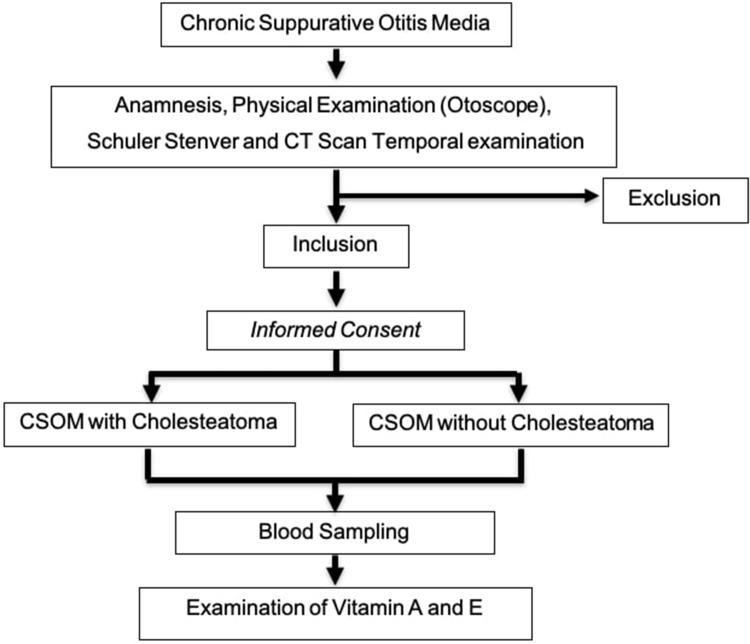
Flow chart of subject.

The study was approved by the Ethical Committee of the Universitas Padjadjaran (LB.02.01/X.6.5/206/2020), the Education and Research Department of the Dr. Hasan Sadikin Hospital (LB.02.01/X.2.2.1/19664/2020) and the Helsinki Declaration principles were implemented to ensure the security, rights, and confidentiality of patients/respondent, while informed consent was obtained from the participants. CSOM patients with or without Cholesteatoma who have not had surgery were recruited from the otology polyclinic within the Department of Otorhinolaryngology-Head and Neck Surgery of Dr. Hasan Sadikin Hospital, Bandung. Following a comprehensive assessment that included history, informed consent, anamnesis, otoscope, Schuler Stenver, and CT Scan Temporal examination and diagnosis, as presented in Figure 1, eligible patients were enrolled for blood sampling. The exclusion criteria for this study were patients without cardiovascular disease, lung disease, metabolic syndrome, liver and kidney disease, patients who had not received vitamin A and E within 1 month, active and passive smokers, as well as pregnant, and all the exclusion criteria based on the anamnesis of the patients.

### Sample Collection

CSOM with or without Cholesteatoma patients had 3 mL of venous blood collected from the antecubital vein into a vacutainer/SST 3.5 mL tube to separate serum and blood cells. The obtained samples were allowed to stand for 30 min at room temperature, followed by centrifugation at 1800–2200 rpm for 10–15 min. The process resulted in a clear, light-yellow layer at the top, which was subsequently analyzed for vitamin A and E levels.

### Quantification of Serum Vitamin A and E

For vitamin A and E determination, 150 μL of serum was transferred into a 1.5 mL tube. Isopropanol (300 μL) and an internal standard (0.75 mg/L acetic retinyl) were added to the tube, and the mixture was vigorously vortexed for 2 min. Following this, the mixture was centrifuged for 15 min at 8500 revolutions per minute. The supernatant was collected, and the determination of vitamin A and E levels was conducted using the high-performance liquid chromatography (HPLC) method.^17^

### Statistics

The primary outcome variables were the serum levels of Vitamin A and E in CSOM patients with and without Cholesteatoma. The data was initially explored using the SPSS (Statistical Product and Service Solution) 25.0 version software for Windows. The Shapiro-Wilk test was employed for data normalization, and the Man Whitney test was used to compare two groups, with median differentiation. Correlations between variables were evaluated using the chi-square test. A statistical significance level of *P* < 0.05 was applied to all analyses.

## Results

The study population was divided into CSOM patients with and without Cholesteatoma, each consisting of 30 participants. Among them, there were 14 males and 16 females in the CSOM with Cholesteatoma group (*P*=0.795). The age range of participants in this group was between 3 and 66 years, with a mean age of 32 (SD = 12.5). Meanwhile, the CSOM patients without Cholesteatoma comprised 13 males and 17 females, with ages ranging from 11 to 66 and with a mean of 33.1 (SD = 12.7, *P*=0.357). The BMI for the group with and without Cholesteatoma ranged from 5 to 17 kg/m^2^ and 4 and 18 kg/m^2^, respectively, with means of 24.9 (SD = 3.2) and 24.8 (SD = 4.1, *P*=0.934) as presented in Table 1.

**Table 1:** Characteristics of Respondents

Characteristics	Group		*P* value*
	CSOM with Cholesteatoma (n = 30)	CSOM without Cholesteatoma (n = 30)	
Sex			
Male	14	13	0.795
Female	16	17	
Age (years)			
<17	3	0	0.357
17–25	9	11	
26–45	13	14	
46–66	5	5	
Mean (SD)	32.0 (12.5)	33.1 (12.7)	
Range	15–55	17–66	
BMI (kg/m^2^)**			
<18.5	5	4	0.934
18.5–23.0	6	5	
23–27.5	2	3	
≥ 27.5	17	18	
Mean (SD)	24.9 (3.2)	24.8 (4.1)	
Range	17.6–32.4	17.1–32.9	

**Notes**: *Based on Chi-square analysis. **World Health Organization (WHO) Asian-population criteria^18^.

**Figure 2:**
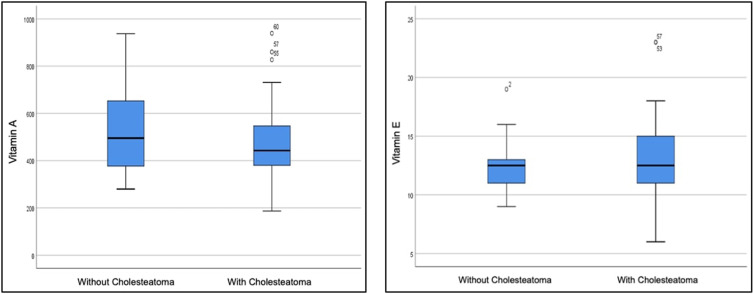
Boxplot differences in serum vitamin A and E levels in the CSOM group with and without cholesteatoma.

In the CSOM patients with Cholesteatoma, serum vitamin A levels ranged from 187 to 939 ug/L, with a mean of 503.77 ug/L, median value of 442.5 ug/L, and SD=173.85, P=0.009. For CSOM patients without Cholesteatoma, the serum vitamin A levels ranged from 280 to 937 ug/L, with a mean of 529.93 ug/L, median value of 496 ug/L, and SD=180.15 (*P*=0.636). The serum vitamin E levels for the first group ranged from 6 to 23 mg/L, with a mean of 12.97 mg/L, median value of 12.5 mg/L, and SD=3.82, *P*=0.036. Meanwhile, for the second group, it ranged from 9 to 19 mg/L, with a mean of 12.6 mg/L, a median value of 12.5 mg/L, and SD=2.22, *P*=0.041 as presented in Table 2. The difference in vitamin A and E serum levels in the CSOM groups is also shown in Figure 2.

**Table 2:** Differences in Vitamin A and E Serum Levels in the CSOM Group with and without Cholesteatoma

Variables	Mean	SD	Median	Range	*P* value*
**Vitamin A (ug/L):**					
CSM with Cholesteatoma	503.77	173.85	442.5	187–939	0.009
CSM without Cholesteatoma	529.93	180.15	495.5	280–937	0.038
**Vitamin E (mg/L):**					
CSM with Cholesteatoma	12.97	3.82	12.5	6–23	0.036
CSM without Cholesteatoma	12.6	2.22	12.5	9–19	0.041

**Notes**: *Analysis using the Mann Whitney test. Vitamin A: Normal (≥700 ug/L), Low (<700 ug/L). Vitamin E: Normal (≥13 mg/L), Low (<13 mg/L).

In this study of CSOM patients without Cholesteatoma, 26 and 4 had low and normal levels of vitamin A, respectively. Among the patients with Cholesteatoma, 25 and 5 had a low and normal level of vitamin A, respectively (OR, 0.769; 95% CI, 0.185–3.198; *P*=1.000). In the first group, the number of patients with a low and normal level of vitamin E was 15 each. The same result was reported in the second (OR, 1.000; 95% CI, 0.363–2.751; *P*=1.000) as presented in Table 3.

**Table 3:** Association of Low Serum Levels of Vitamin A and E with the Occurrence of CSOM with and without Cholesteatoma

Characteristic	CSOM without Cholesteatoma n=30 n (%)	CSOM with Cholesteatoma n=30 n (%)	*P-*value*	OR (95% CI OR)
**Vitamin A**				
Normal	4 (13.3)	5 (16.7)	1.000	0.769 (0.185–3.198)
Low	26 (86.7)	25 (83.3)		
**Vitamin E**				
Normal	15 (50)	15 (50)	1.000	1.000 (0.363–2.751)
Low	15 (50)	15 (50)		

**Notes**: *Analysis using Chi-Square test. Vitamin A: Normal (≥700 ug/L), Low (<700 ug/L). Vitamin E: Normal (≥13 mg/L), Low (<13 mg/L).

## Discussion

In this study, observations showed that vitamin A and E levels in CSOM with cholesteatoma were lower and higher, respectively, as presented in Figure 2. Following meticulous statistical analysis, it was established that both vitamin A and vitamin E levels did not exhibit significant differences between the two groups, yielding p-values of 1.000 for both. These results may be attributed to variations in sample sizes and potential gender disparities. Tria et al’s study (2020) reported a 57% occurrence of CSOM cases with cholesteatoma in women, while^19^ Saitabau et al discovered a gender-leaning trend in CSOM sufferers.^20^ Martanegara et al (2020) noted a significant male predominance at 66.84%.^21^ Conversely, Nugroho et al (2013) observed a twofold higher prevalence in women compared to men.^22^ These studies suggested no significant gender-based disparities, aligning with the results as no significant distinctions were observed based on gender characteristics.

Gaurano and Johaarjy reported that most cholesteatoma patients were aged between 20 and 35.^23^ Similarly, Pratama et al conducted a study at Sanglah General Hospital, reporting that 43.2%, 18.9%, and 18.6% of CSOM cases occurred in adults, elderly, and teenagers.^24^ Similar to Wilsen’s study at H Adam Malik Hospital in Medan, 56.5% of the patients were aged over 18.^25^ Martanegara et al (2020) also observed that most (64.72%) cases were in the age range of 21 −30 years. In this study, a similar trend was identified in both CSOM with and without cholesteatoma, with the highest incidence occurring in the age range of 17 to 55, peaking between 15 and 25 years. This age group appears particularly susceptible to CSOM, likely due to their productive age and potentially lower attention to hygiene, sanitation, as well as overall health.^21^ Natarajan et al’s study (2014) reported different results, with CSOM most commonly occurring in children under 10 years old. This could be attributed to inadequate diet and nutrition in the age group, which weakens the immune system, damages mucosal tissues, and hinders growth and development in children.^10^

Various factors may influence the association between serum levels of vitamins A and E and CSOM with cholesteatoma. Epithelial cell metaplasia is a common occurrence in organs primarily composed of ciliated columnar epithelial cells, such as the eustachian tube. Therefore, vitamin deficiencies may be closely linked to eustachian tube dysfunction, potentially triggering inflammation in the middle ear. The formation of cholesteatoma involves various inflammatory mediators, including proteins, peptides, glycoproteins, cytokines, arachidonic acid metabolites (prostaglandins and leukotrienes), nitric oxide, and free radicals.^26^

To prevent the reaction of excessive free radical formation, the body inhibits this process through the use of antioxidants.^14^ In some instances, the endogenous antioxidant is not sufficient to reduce excessive oxidative stress, necessitating the application of external antioxidants.^27^ Vitamins A and E serve as secondary or exogenous antioxidants, effectively inhibiting and neutralizing oxidation reactions.^28^,^29^ Study conducted by Baysal et al identified a significant increase in serum oxidant status and oxidative stress index among CSOM patients. Furthermore, changes in diet and nutritional intake can impact the levels of these vitamins in the blood.^16^ A study conducted in Nepal showed that high-dose vitamin A supplementation in children significantly reduces the risk of hearing loss due to suppurative ear infections in adolescents or adults. In this context, Vitamin A plays a role in cell differentiation and epithelial proliferation.^30^,^31^ Additionally, Vitamin E, being a fat-soluble antioxidant, effectively inhibits peroxyl radicals and halts the oxidation of polyunsaturated fatty acids (PUFA).^28^ According to a study by Greaves, et al, an advantage of vitamin A is its effectiveness at low concentrations of oxygen. Therefore, it can complement the antioxidant properties of vitamin E that is effective at high concentrations.^29^

In this study, it was observed that patients with low levels of vitamins A and E did not increase the risk of CSOM with cholesteatoma. Furthermore, the mean serum level of vitamin A was slightly lower in this group, but not significantly different. The study by Arulselvan et al stated that otitis media is a manifestation of vitamin A deficiency.^31^ Cemek et al (2015) also reported that patients with otitis media showed retinol levels of 22.78 ± 2.01 µg/dL and were lower than the control group of 32.81 ± 2.13 µg/dL (p<0.001).^32^ Lasisi et al reported that deficiency of vitamin A has a potential effect for chronic suppurative otitis media and recommended vitamin A and E treatment to reduce the risk of suppurative otitis media.^33^

Vitamin A, E, and their derivatives were needed to maintain epithelial mucociliary and mucus secretion via specific nuclear receptors. Deficiency of those vitamins induced the replacement of mucociliary epithelium and resulted in the alteration of mucus secretion.^30^ Vitamin A deficiency was also associated with a deficiency in immunomodulatory functions and induced an up-regulation of the T helper 1 cell. The study by Lasisi showed that individuals with persistent otorrhea had low serum levels of both vitamin A and E. This suggested a potential role for these vitamins in the pathophysiology of CSOM.^30^,^33^ In addition to vitamin A, tocopherol (vitamin E) is one of the most important nonenzymatic antioxidant defense systems in inflammatory processes, which occur in several diseases including chronic otitis media (COM) and CSOM. Antioxidant deficiencies can lead to oxidative stress, which can damage the ciliary structure of cell membranes in the middle ear, hence, prolonging inflammation and potentially contributing to chronicity.^13^,^16^ Furthermore, Aydogan et al also reported that as antioxidants defence systems, vitamin A and E could prevent injuries that lead to middle ear inflammation, especially due to the production of free radicals.^34^

In this study, the opposite results were obtained, the mean vitamin E levels tended to be higher in the group with cholesteatoma, but were not significantly different. These results aligned with Aydogan et al’s study, which observed higher levels of vitamin E in patients with otitis media with effusion and adenoid hyperplasia compared to the control group. It is important to note that these outcomes can be influenced by several factors such as adequate health-care facilities, socio-economic status of the community, and good nutrition.^34^ As an antioxidant, tocopherol contains hydrogen ions, which are effective and quickly react with several free radicals, preventing them from damaging cell membranes and other components. Vitamin E deficiency tends to increase the risk of free radicals, which are the pathogenesis of chronic inflammation in CSOM with cholesteatoma.^28^,^32^,^35^

## Study Limitation

The primary focus should be on the number of patients and methods of measuring the levels of these vitamins in cholesteatoma and middle ear mucosa, then socioeconomic status, nutrition intake, and zinc levels were not specifically examined to distinguish patients with vitamin A deficiency due to a lack of zinc and vitamin A intake. Zinc is needed to aid the absorption of vitamin A and the formation of retinol-binding protein (RBP), namely intravascular transport protein. This function is to transport retinol from the liver to the bloodstream and other organs, thereby preventing the loss of vitamin A through filtration in the kidney.^34^

## Conclusion

In conclusion, the role of Vitamin A and E levels had no impact on the occurrence of CSOM with or without cholesteatoma. Therefore, further clinical studies especially number of patients and methods of measuring the levels of these vitamins need to be conducted for more representative results.

## Abbreviation

AOM, acute otitis media; OME, otitis media with effusion; CSO, chronic suppurative otitis media; ROS, reactive oxygen species; CTA, computerized tomography; SSTS, serum separation tubes; HPL, high-performance liquid chromatography; SPSS, Statistical Product and Service Solution; BMI, body mass index; SD, standard deviation; OR, odds ratio.
